# Supplementary material for: Longitudinal plasma metabolomics of aging and sex
Source: Aging (Albany NY). 2019 Feb 24;11(4):1262–82. doi: 10.18632/aging.101837 (PMC6402508; doi:10.18632/aging.101837)
Supplement: Supplementary Figures [file aging-11-101837-s002.pdf]

SUPPLEMENTARY FIGURES

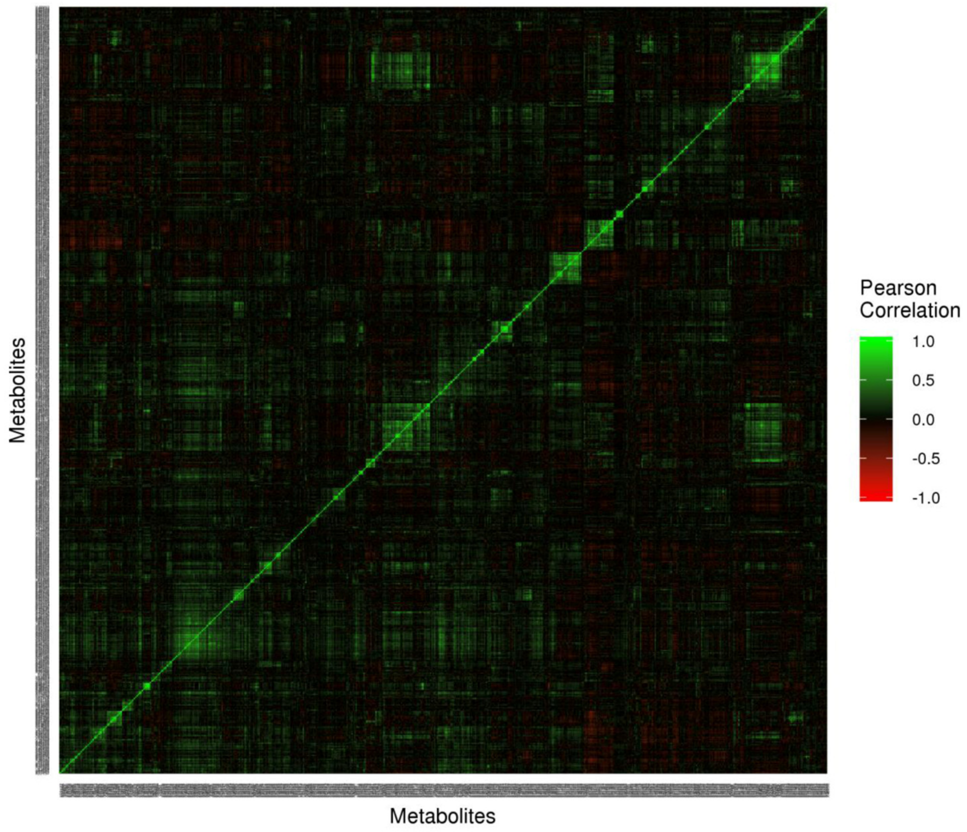

Figure S1. Heatmap of metabolite correlations. Pearson correlations (r) are displayed and hierarchical clustering was used to sort metabolites. Clusters of strong positive correlations are largely between amino acid–amino acid and between lipid–lipid (i.e., correlations between super pathway had weaker correlations). Negative correlations are less common and all have an $r \geq -0.62$.

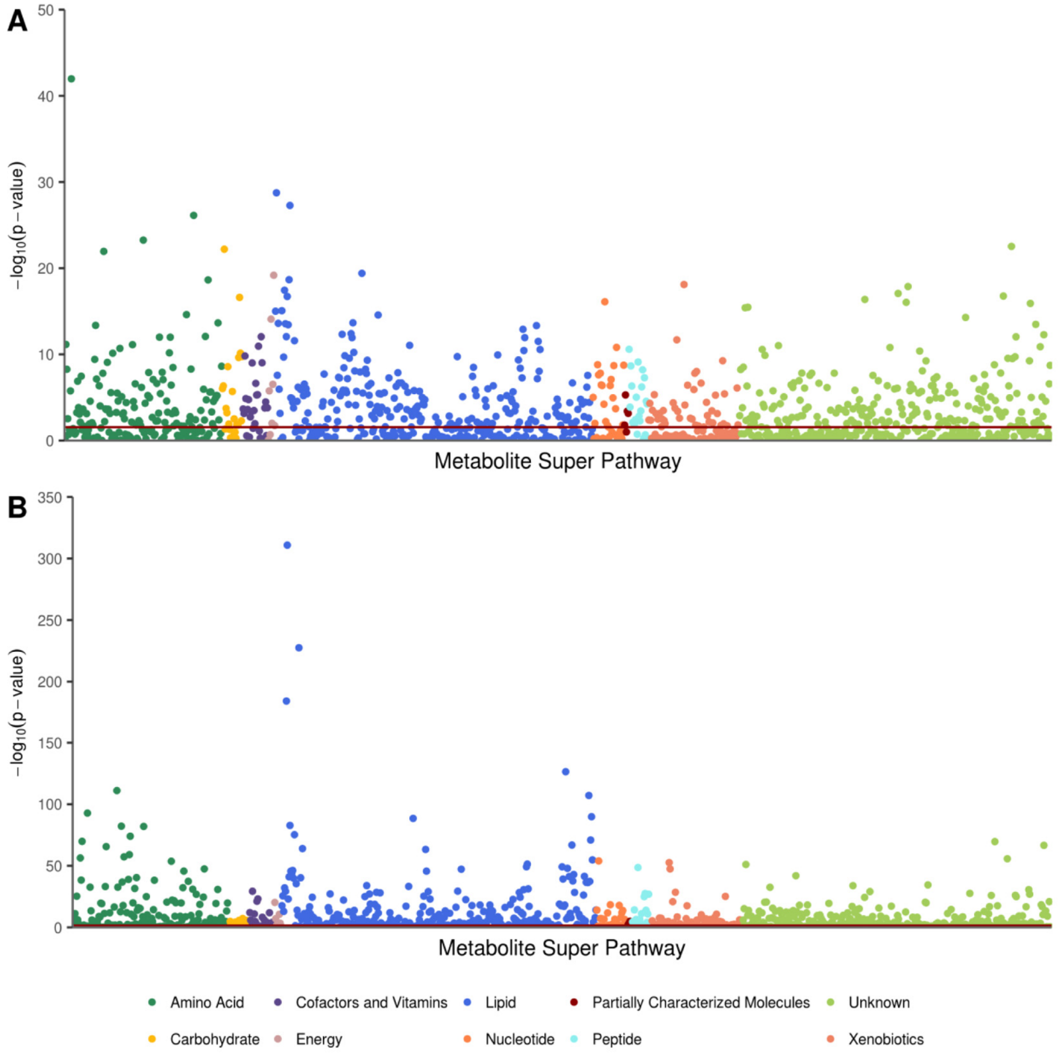

Figure S2. Manhattan plots of metabolome-wide associations results. (**A**) Age significantly influenced 623 metabolites. (**B**) Sex significantly influenced 695 metabolites. Both sets of results use a Benjamini-Hochberg adjusted p-value threshold (red horizontal line).

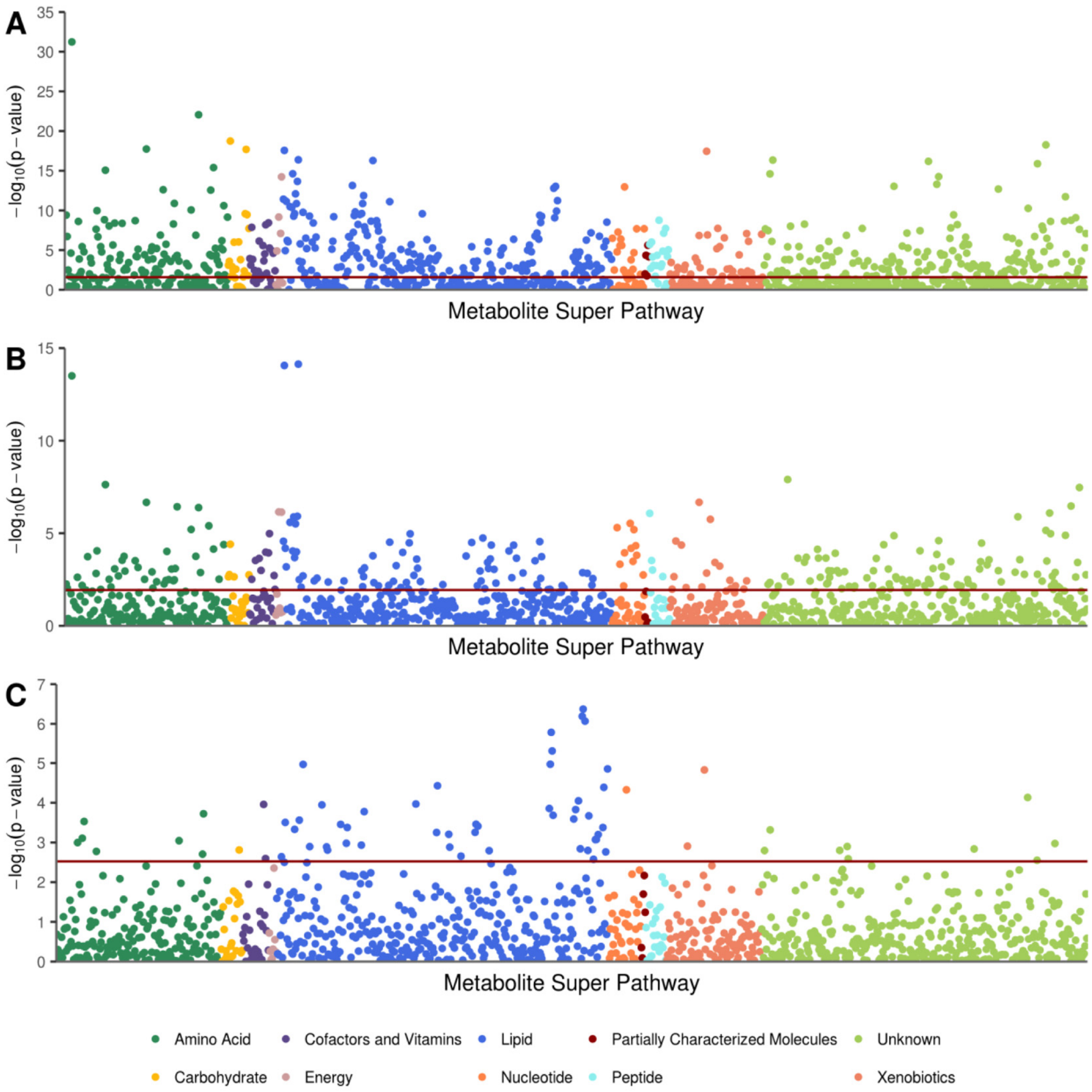

Figure S3. Manhattan plots of metabolome-wide associations results. (A) Age significantly influenced 565 metabolites in women. ()) Age significantly influenced 255 metabolites in men. ()) Trajectories of 68 metabolites significantly differ by sex. Each set of results uses a Benjamini-Hochberg adjusted p-value threshold (red horizontal line).

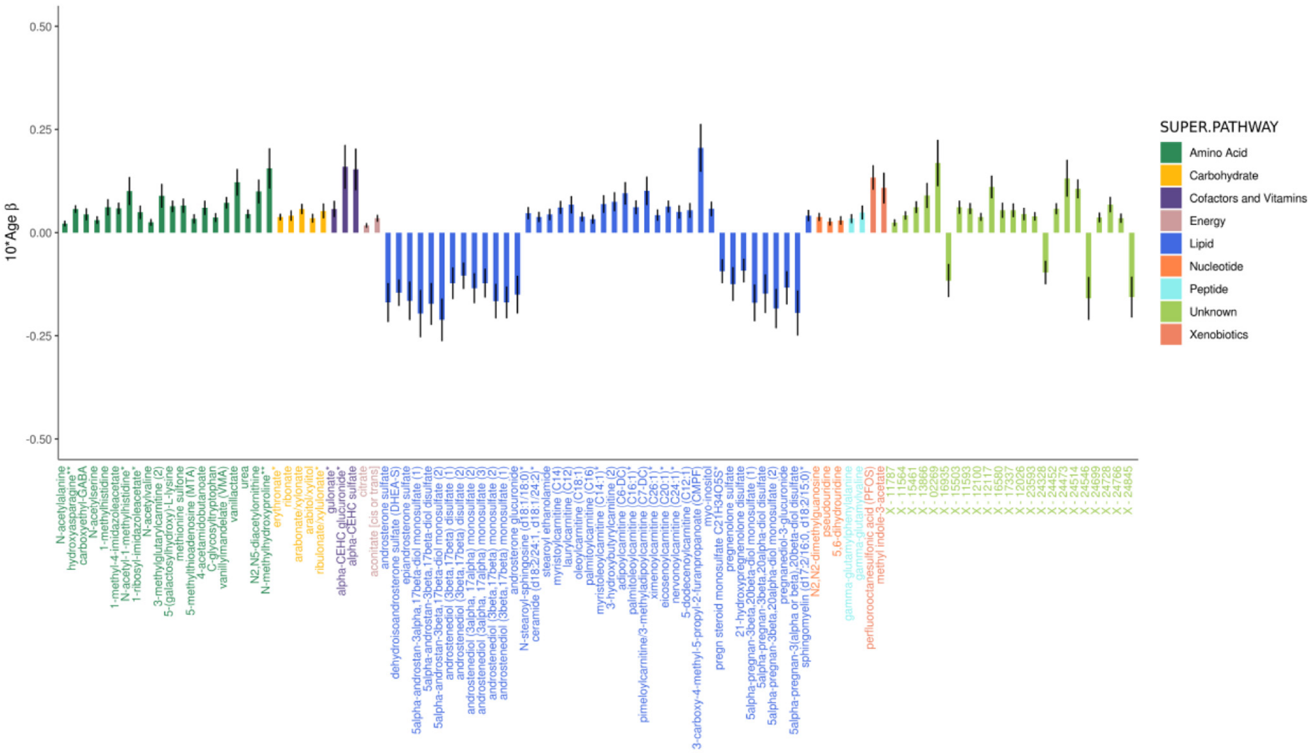

Figure S4. Age stratified by sex: Adjusted effects of a 10-year increase in age on the top 100 metabolites most strongly influenced by age in women. Positive values indicate the amount a metabolite increased over 10 years, whereas negative values indicate the amount a metabolite decreased over 10 years. Black vertical lines indicate standard errors.

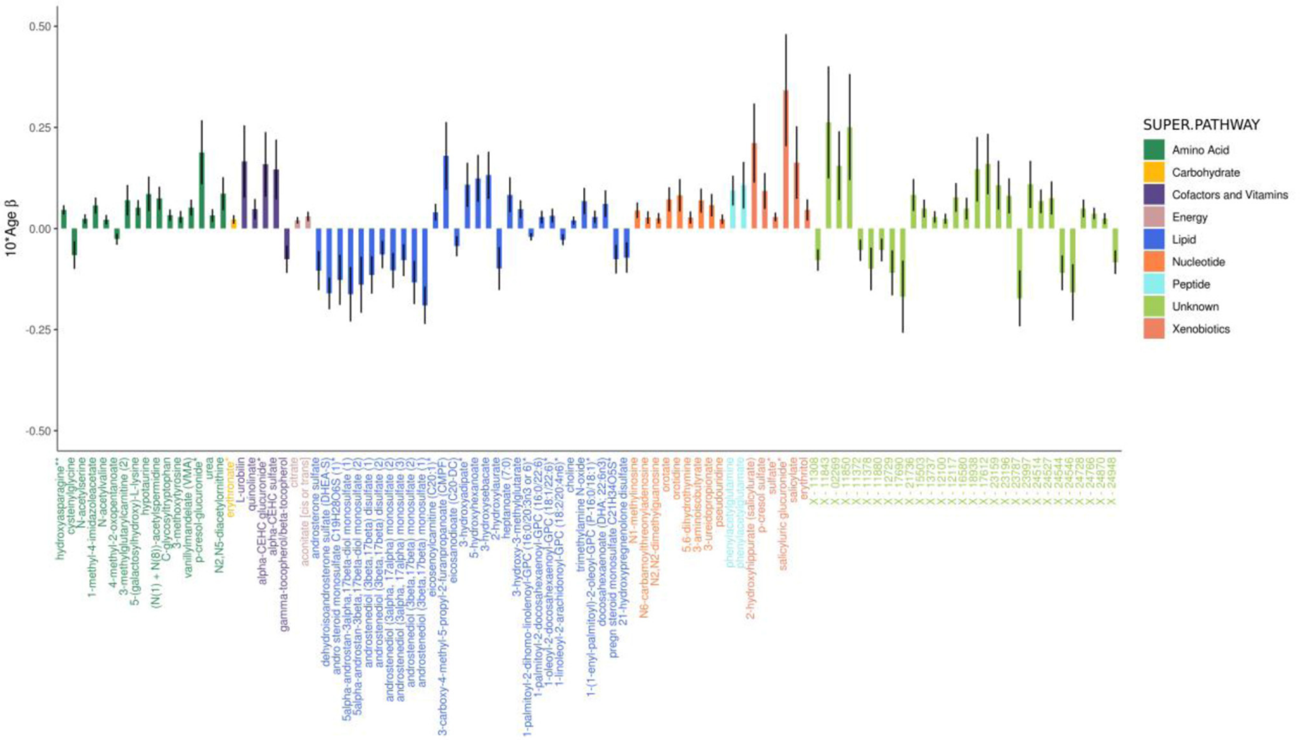

Figure S5. Age stratified by sex: Adjusted effects of a 10-year increase in age on the top 100 metabolites most strongly influenced by age in men. Positive values indicate that the metabolite increased with 10 years of age, whereas negative values indicate that the metabolite decreased with 10 years of age. Black vertical lines indicate standard errors.

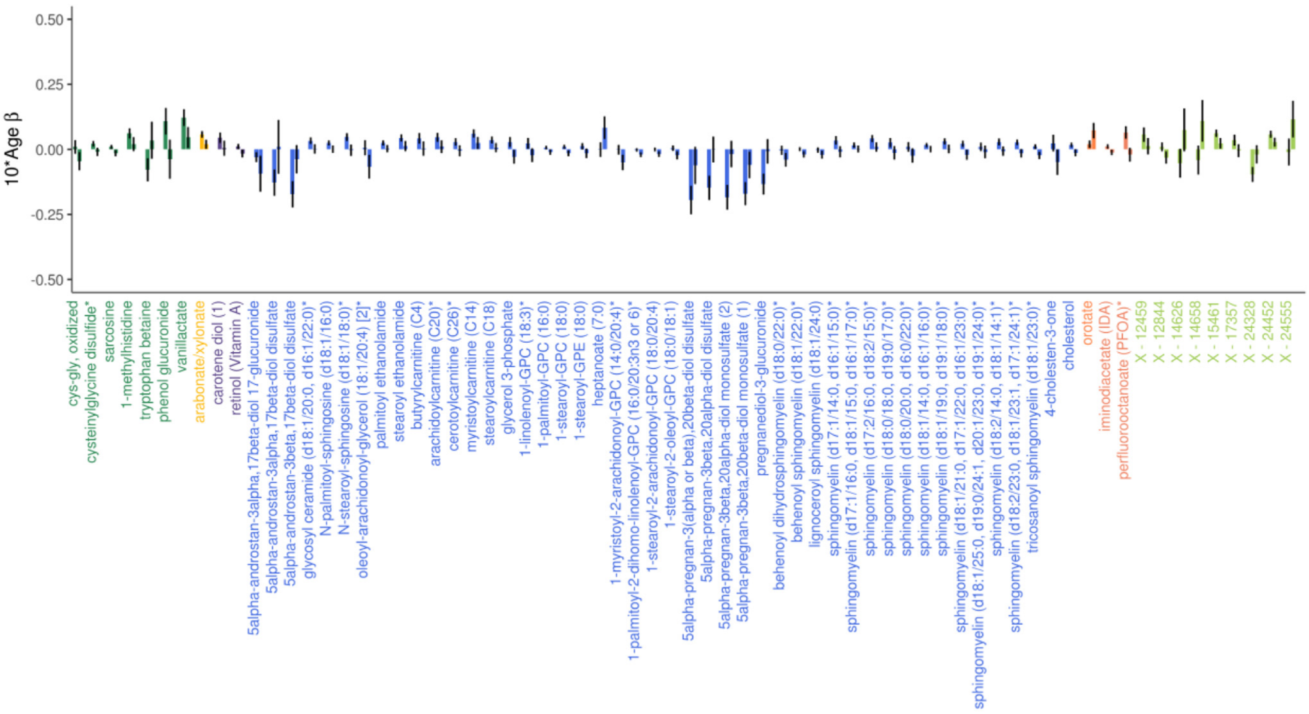

Figure S6. Adjusted effects of a 10-year increase in age on the 68 metabolites with trajectories that significantly differ by sex. For each metabolite, the bar on the left represents the change in metabolite level over 10 years in women, whereas the bar on the right represents the change in metabolite level over 10 years in men. Black vertical lines indicate standard errors.

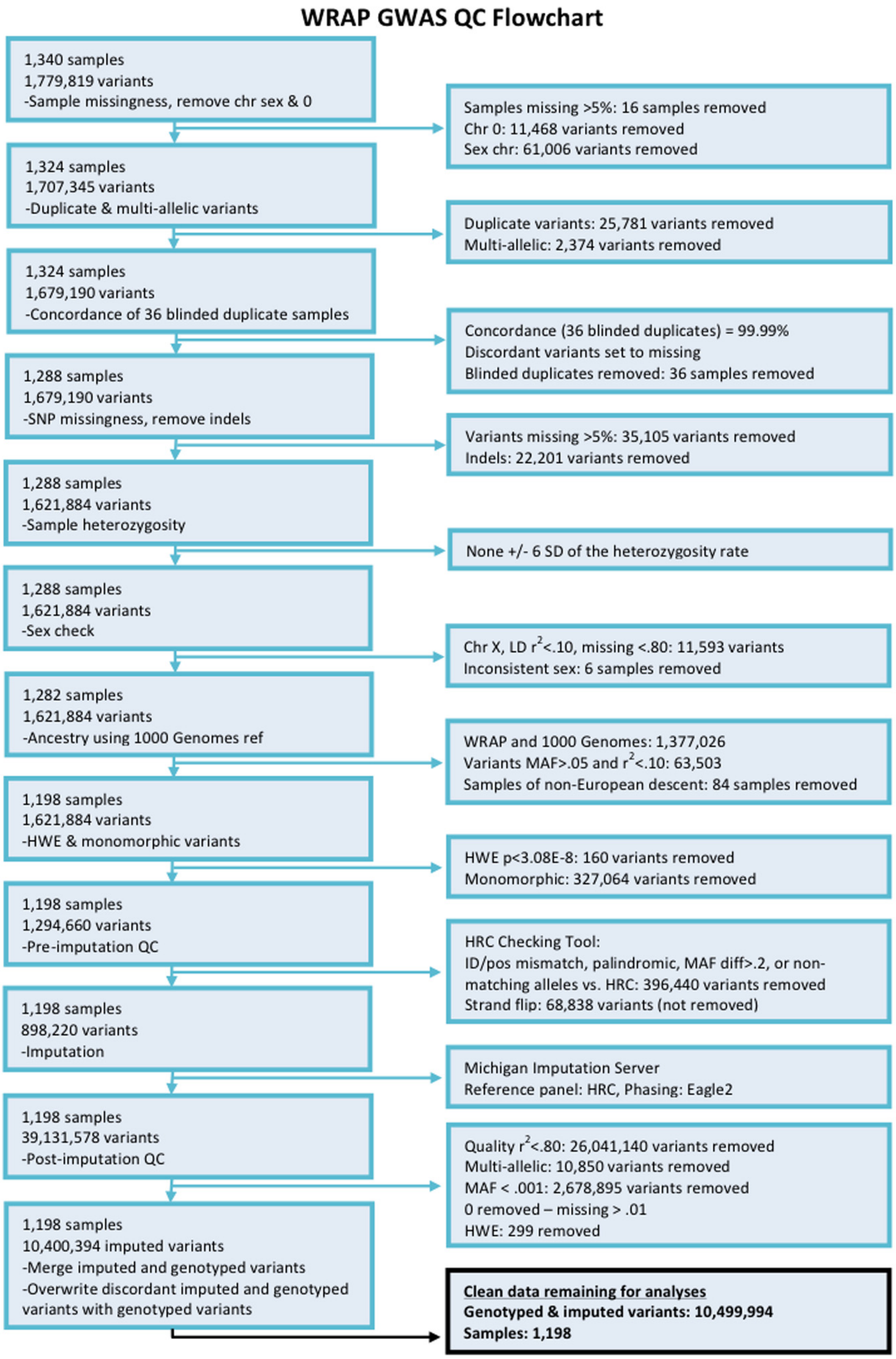

Figure S7. GWAS QC Flowchart.
